# 1948. Rise of *Candida auris* Infections Worldwide and Trends on the Activity of Fosmanogepix and Comparator Agents against *C. auris* Causing Invasive Infections

**DOI:** 10.1093/ofid/ofad500.102

**Published:** 2023-11-27

**Authors:** Cecilia G Carvalhaes, Michael D Huband, Paul Rhomberg, Beth Hatch, Mariana Castanheira

**Affiliations:** JMI Laboratories, North Liberty, IA; JMI Laboratories, North Liberty, IA; JMI Laboratories, North Liberty, IA; JMI Laboratories, North Liberty, IA; JMI Laboratories, North Liberty, IA

## Abstract

**Background:**

*Candida auris* (CAU) epidemiology is evolving rapidly. Manogepix is the active moiety of the novel prodrug antifungal fosmanogepix (FMGX), and has broad-spectrum activity against yeasts and moulds, including CAU. Fluconazole (FLC) resistance (R) is common in CAU and R to amphotericin B (AMB) and echinocandins (ECHs) is documented. Global trends on CAU frequency and susceptibility (S) rates to MGX and comparators were evaluated through the MGX Surveillance Program.

**Figure:**
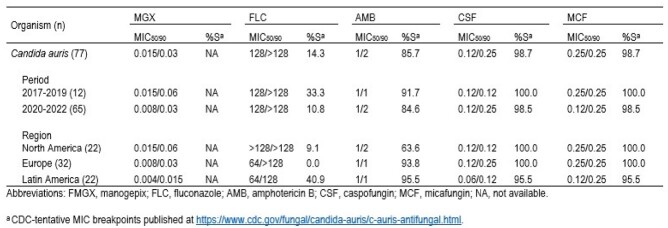

**Methods:**

A total of 77 CAU collected (1/patient) in 2017–2022 from 12 medical centers in North America (NA; *n*=22; 6 centers), Europe (EU; *n*=32; 4 centers), Asia-Pacific (AP; *n*=1; 1 centers), and Latin America (LA, *n*=22; 1 centers) were identified by MALDI-TOF MS and tested by CLSI broth microdilution. CDC tentative breakpoints (BP) were applied.

**Results:**

CAU were mainly recovered from bloodstream infections (68.8%). Twelve CAU isolates were recovered in 2017–2019 (0.4% of all *Candida*) and 65 in 2020–2022 (2.2%; Table). CAU incidence spiked between these two time periods in NA (6 to 16), EU (0 to 32), and LA (6 to 16). MGX showed potent activity against CAU overall (MIC_50/90_, 0.015/0.03 mg/L), inhibiting all isolates at ≤ 0.06 mg/L. MGX was 8-fold more active than ECHs (MIC_50/90_ range, 0.12–0.25/0.25–0.5 mg/L), which inhibited all but 1 isolate at their respective BPs (98.7%S). FLC-R rates rose from 66.7% in 2017–2019 to 89.2% in 2020–2022, but varied among regions from 100.0% in EU to 90.9% in NA and 59.1% in LA. Only 1 AP isolate was recovered in 2022. This isolate exhibited an MGX MIC of 0.015 mg/L and was S to ECH and AMB but R to FLC. A decrease in AMB S rates was noted overall (from 91.7% to 84.6%). The lower AMB S rate was observed in NA isolates (63.6%S) rather than EU (93.8%S) and LA (95.5%S) isolates. The FLC-R and AMB-R phenotypes were noted in 11 isolates (14.3%; 8 from NA; all but 1 from 2020–2022). MGX (MIC_50/90_, 0.03/0.06 mg/L) and ECH (100%S) remained active against those highly R isolates.

**Conclusion:**

In the MGX Surveillance Program, recovery of CAU increased considerably over time in all regions except AP. The rise of the FLC-R and AMB-R phenotypes is worrying, especially in NA. MGX and ECH remained active against CAU regardless of the resistant phenotype or study period.

**Disclosures:**

**Cecilia G. Carvalhaes, MD, PhD**, AbbVie: Grant/Research Support|bioMerieux: Grant/Research Support|Cipla: Grant/Research Support|CorMedix: Grant/Research Support|Melinta: Grant/Research Support|Pfizer: Grant/Research Support **Michael D. Huband, BS**, BARDA: This study has been funded in part by BARDA under Contract No. 75A50120C00001.|Entasis: Grant/Research Support|Paratek: Grant/Research Support|Pfizer: Grant/Research Support **Paul Rhomberg, BS, MT(ASCP)**, bioMerieux: Grant/Research Support|Melinta: Grant/Research Support|Pfizer: Grant/Research Support **Beth Hatch, BS, MT(ASCP)**, Pfizer: Grant/Research Support **Mariana Castanheira, PhD**, AbbVie: Grant/Research Support|Basilea: Grant/Research Support|bioMerieux: Grant/Research Support|Cipla: Grant/Research Support|CorMedix: Grant/Research Support|Entasis: Grant/Research Support|Melinta: Grant/Research Support|Paratek: Grant/Research Support|Pfizer: Grant/Research Support|Shionogi: Grant/Research Support

